# Calorie restriction reduces biomarkers of cellular senescence in humans

**DOI:** 10.1111/acel.14038

**Published:** 2023-11-14

**Authors:** Zaira Aversa, Thomas A. White, Amanda A. Heeren, Cassondra A. Hulshizer, Dominik Saul, Xu Zhang, Anthony J. A. Molina, Leanne M. Redman, Corby K. Martin, Susan B. Racette, Kim M. Huffman, Manjushri Bhapkar, Sundeep Khosla, Sai Krupa Das, Roger A. Fielding, Elizabeth J. Atkinson, Nathan K. LeBrasseur

**Affiliations:** ^1^ Robert and Arlene Kogod Center on Aging, Mayo Clinic Rochester Minnesota USA; ^2^ Department of Physical Medicine and Rehabilitation Mayo Clinic Rochester Minnesota USA; ^3^ Department of Quantitative Health Sciences Mayo Clinic Rochester Minnesota USA; ^4^ Department of Trauma and Reconstructive Surgery Eberhard Karls University Tübingen, BG Trauma Center Tübingen Tübingen Germany; ^5^ Department of Medicine University of California San Diego La Jolla California USA; ^6^ Pennington Biomedical Research Center Baton Rouge Louisiana USA; ^7^ College of Health Solutions Arizona State University Phoenix Arizona USA; ^8^ Program in Physical Therapy Washington University School of Medicine St. Louis Missouri USA; ^9^ Duke Clinical Research Institute and Molecular Physiology Institute, School of Medicine Durham North Carolina USA; ^10^ Division of Endocrinology Mayo Clinic College of Medicine Rochester Minnesota USA; ^11^ Energy Metabolism Team, Jean Mayer USDA Human Nutrition Research Center on Aging Tufts University Boston Massachusetts USA; ^12^ Nutrition, Exercise Physiology and Sarcopenia Laboratory, Jean Mayer USDA Human Nutrition Research Center on Aging Tufts University Boston Massachusetts USA; ^13^ Paul F. Glenn Center for the Biology of Aging at Mayo Clinic Rochester Minnesota USA

**Keywords:** aging, biomarkers, CALERIE™, caloric restriction, inflammation, metabolism, senescence‐associated secretory phenotype

## Abstract

Calorie restriction (CR) with adequate nutrient intake is a potential geroprotective intervention. To advance this concept in humans, we tested the hypothesis that moderate CR in healthy young‐to‐middle‐aged individuals would reduce circulating biomarkers of cellular senescence, a fundamental mechanism of aging and aging‐related conditions. Using plasma specimens from the Comprehensive Assessment of Long‐term Effects of Reducing Intake of Energy (CALERIE™) phase 2 study, we found that CR significantly reduced the concentrations of several senescence biomarkers at 12 and 24 months compared to an ad libitum diet. Using machine learning, changes in biomarker concentrations emerged as important predictors of the change in HOMA‐IR and insulin sensitivity index at 12 and 24 months, and the change in resting metabolic rate residual at 12 months. Finally, using adipose tissue RNA‐sequencing data from a subset of participants, we observed a significant reduction in a senescence‐focused gene set in response to CR at both 12 and 24 months compared to baseline. Our results advance the understanding of the effects of CR in humans and further support a link between cellular senescence and metabolic health.

## Graphical abstract

Using samples from the CALERIE™ study, we demonstrate that calorie restriction (CR) significantly reduced the concentrations of several circulating biomarkers of cellular senescence compared to an ad libitum diet. Changes in biomarker concentrations were important predictors of the change in HOMA‐IR and insulin sensitivity index, and the change in resting metabolic rate residual. Using adipose tissue RNA‐sequencing data from a subset of participants, we also observed a significant reduction in a senescence‐focused gene set in response to CR. Our results advance the understanding of the effects of CR in humans and further support a link between cellular senescence and metabolic health.

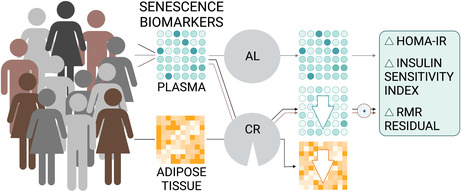

**Abbreviations**
AL: ad libitum
BMI: body mass index
CALERIE^TM^
: Comprehensive Assessment of Long‐term Effects of Reducing Intake of Energy
CR: calorie restriction
GBM: gradient boosting machine learning
GSEA: gene set enrichment analysis
HOMA‐IR: homeostatic model assessment of insulin resistance
ITT: intention‐to‐treat
NES: normalized enrichment score
RMR: resting metabolic rate
SASP: senescence‐associated secretory phenotype

## 1 INTRODUCTION

Compelling evidence from a wide range of animal studies suggests that calorie restriction (CR) with adequate nutrient intake is a promising strategy to extend lifespan and delay the onset of several age‐related chronic diseases (Balasubramanian et al., 2017). In humans, the Comprehensive Assessment of Long‐term Effects of Reducing Intake of Energy (CALERIE™) has been the most rigorous study to investigate the effects of CR. Following Phase 1, a series of three pilot studies to inform study design (Das et al., 2007; Heilbronn et al., 2006; Racette et al., 2006), Phase 2 of CALERIE™ was a 2‐year, multicenter, randomized controlled trial in healthy non‐obese young‐to‐middle‐aged individuals to examine the safety and effects of moderate CR compared to an ad libitum (AL) diet on predictors of longevity, disease risk factors, and quality of life (Ravussin et al., 2015). Although the average CR attained over the 2 years was ~12% rather than the prescribed 25%, the intervention was deemed safe and effective in promoting cardiometabolic risk reduction (Huffman et al., 2022; Kraus et al., 2019; Ravussin et al., 2015). The long‐term implications of the intervention on healthspan and longevity remain to be established, but the biospecimens collected during the CALERIE™ study represent a unique resource to explore the influence of CR on the biology of aging in humans.

Preclinical studies suggest that CR may exert its beneficial effects by delaying, or potentially preventing, fundamental *hallmarks* of aging (Green et al., 2022; López‐Lluch & Navas, 2016). Cellular senescence, defined as a state of permanent proliferation arrest, is one such hallmark triggered by various forms of damage and stress (Di Micco et al., 2021). Senescent cells progressively accumulate in various tissues with advancing age and, through the secretion of bioactive molecules collectively referred as senescence‐associated secretory phenotype (SASP) (Coppé et al., 2010), can exert local and systemic deleterious effects such as contributing to chronic inflammation (Acosta et al., 2013; Xu et al., 2015, 2018). Preclinical studies suggest that interventions targeting senescent cells positively affect multiple aspects of health (Zhang et al., 2022), including measures of metabolic function such as glucose tolerance and insulin sensitivity (Palmer et al., 2022). Moreover, experimental studies in mice and rats have reported that CR reduces molecular features of cellular senescence in various tissues (Fontana et al., 2018; Krishnamurthy et al., 2004; Mau et al., 2020; Ning et al., 2013; Wang et al., 2010), but the influence of CR on this hallmark of aging in humans is still poorly understood.

To gain new insights, we studied plasma samples obtained at different timepoints from CALERIE™ phase 2 trial participants to test the hypothesis that CR would lower the circulating levels of candidate senescence biomarkers to a great extent than an AL diet. Although not unique to senescent cells, the biomarker panel used here represents the diversity of cytokines, chemokines, matrix remodeling proteins, and growth factors robustly secreted by several human cell types when driven to senesce in vitro and shown to be detectable and increase in the blood of humans with advanced chronological age or clinical evidence of advanced biological age (e.g., frailty or functional decline), consistent with an increase in senescent cell burden (Aversa et al., 2023; Basisty et al., 2020; Fielding et al., 2022; Schafer et al., 2020; Tanaka et al., 2018, 2020). Moreover, most of the measured biomarkers are represented in senescence‐focused gene sets (Coppé et al., 2010; Saul et al., 2022) and/or their circulating concentrations are elevated in mouse models with high senescent cell burden (Englund et al., 2023; Yousefzadeh et al., 2020). Using machine learning, we also examined whether the changes in circulating senescence‐related biomarkers predicted the longitudinal changes in parameters of metabolic health.

## 2 RESULTS

### 2.1 Study participants

For Phase 2 of the CALERIE™ study participants were randomized 2:1 to either a 25% CR diet or an AL diet for 2 years. We studied the clinical data and plasma samples of 199 participants (CR = 128, AL = 71) who had stored specimens available from at least two timepoints. Participants were predominantly white (76%) and female (71%). Baseline demographic and clinical characteristics were similar between CR and AL groups and are summarized in Table 1.

**TABLE 1:** Demographic characteristics at baseline by treatment group.

	Ad libitum group *n* = 71	Calorie restriction group *n* = 128	Total *n* = 199	Between‐ group *p*‐value
Age, years	37.99 (6.97)	38.27 (7.27)	38.17 (7.15)	0.740^a^
Sex				
Female	50 (70.4%)	91 (71.1%)	141 (70.9%)	0.920^b^
Male	21 (29.6%)	37 (28.9%)	58 (29.1%)	
Ethnicity				
White	53 (74.6%)	99 (77.3%)	152 (76.4%)	0.625^b^
African American	11 (15.5%)	14 (10.9%)	25 (12.6%)	
Other	7 (9.9%)	15 (11.7%)	22 (11.1%)	
BMI, Kg/m^2^	25.17 (1.65)	25.16 (1.73)	25.17 (1.70)	0.918^a^
BMI stratum				
Normal weight	34 (47.9%)	61 (47.7%)	95 (47.7%)	0.975^b^
Overweight	37 (52.1%)	67 (52.3%)	104 (52.3%)	
Sex/BMI stratum				
Female/normal weight	29 (40.8%)	52 (40.6%)	81 (40.7%)	0.999^b^
Female/overweight	21 (29.6%)	39 (30.5%)	60 (30.2%)	
Male/normal weight	5 (7.0%)	9 (7.0%)	14 (7.0%)	
Male/overweight	16 (22.5%)	28 (21.9%)	44 (22.1%)	

*Note*: Continuous variables expressed as mean (SD); categorical variables expressed as n (%).

Abbreviation: BMI, body mass index.

^a^ Kruskal–Wallis rank sum test.

^b^ Pearson's chi‐squared test.

### 2.2 Calorie restriction reduces circulating concentrations of senescence biomarkers in humans

**FIGURE 1:**
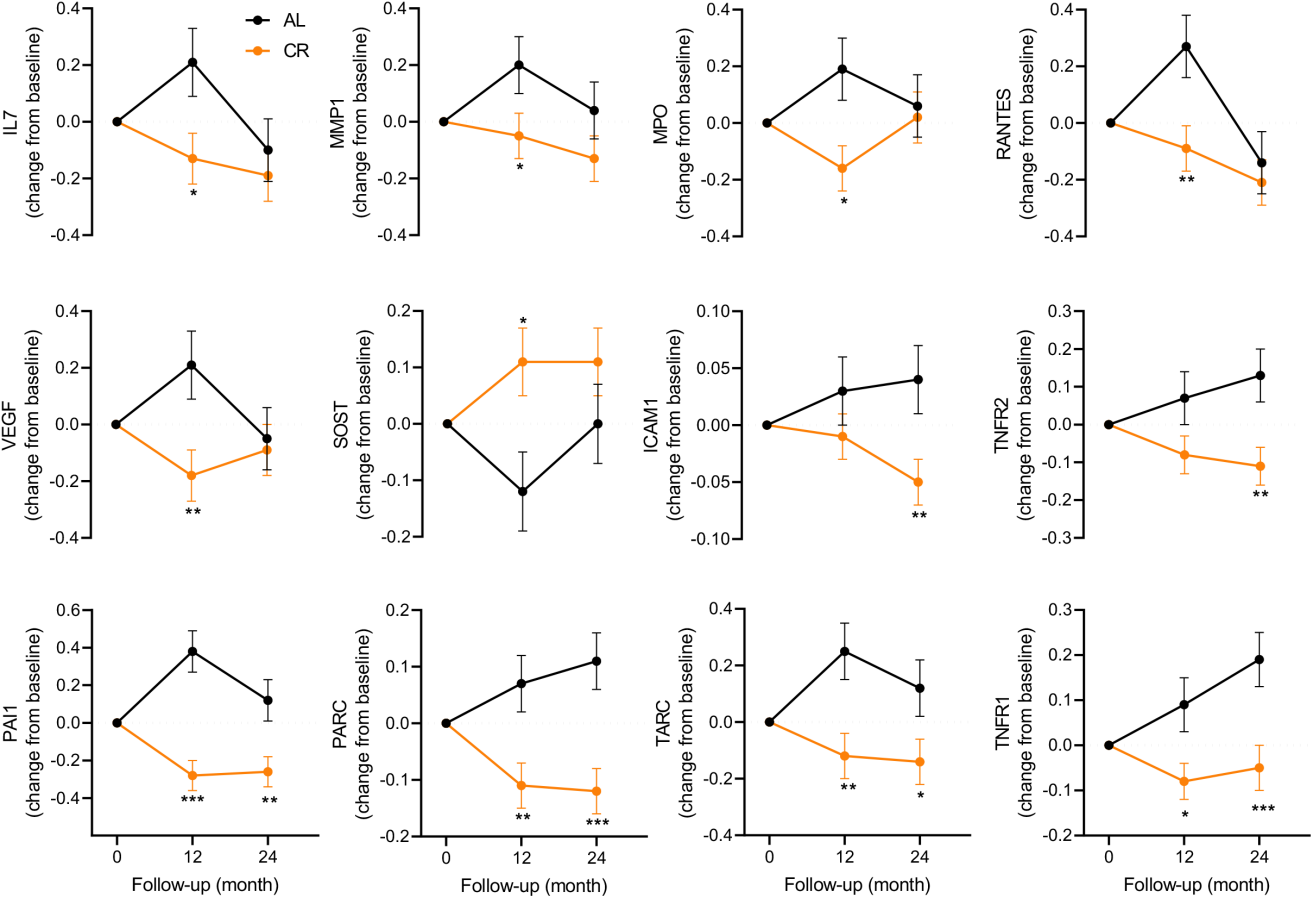
Changes in senescence‐associated biomarkers from baseline to 12‐ and 24‐month timepoints. The changes at 12 months and 24 months were evaluated by mixed effects repeated measures analysis; the symbols *, ** and *** denote *p* < 0.05, 0.01 and 0.001, respectively, between the ad libitum (AL) and the calorie restriction (CR) groups.

The concentrations of 28 candidate senescence biomarkers were quantified in baseline, 12‐month, and 24‐month plasma samples (Table S1) (Schafer et al., 2020). The change in protein levels from baseline to 12 months and baseline to 24 months were compared between CR and AL groups after adjusting for baseline values, sex, BMI stratum (normal weight, overweight), and study site. In comparison to AL, CR at 12 months significantly reduced the circulating concentrations of 9 senescence biomarkers—IL7, MMP1, MPO, PAI1, PARC, RANTES, TARC, TNFR1, and VEGF—and increased the concentration of only one biomarker—SOST— (Figure 1; Table S2). At 24 months, reduced concentrations of PAI1, PARC, TARC, and TNFR1 persisted in the CR participants compared to the AL participants, ICAM1 and TNFR2 were also reduced, while RAGE was increased.

### 2.3 Changes in senescence biomarkers are associated with changes in measures of metabolic health

We next examined the extent to which the longitudinal changes in senescence biomarkers were associated with changes in the homeostatic model assessment of insulin resistance (HOMA‐IR), an exploratory metabolic outcome evaluated in the CALERIE™ study that improved in CR compared to AL participants at both the 12‐ and 24‐month timepoints (Kraus et al., 2019).

**FIGURE 2:**
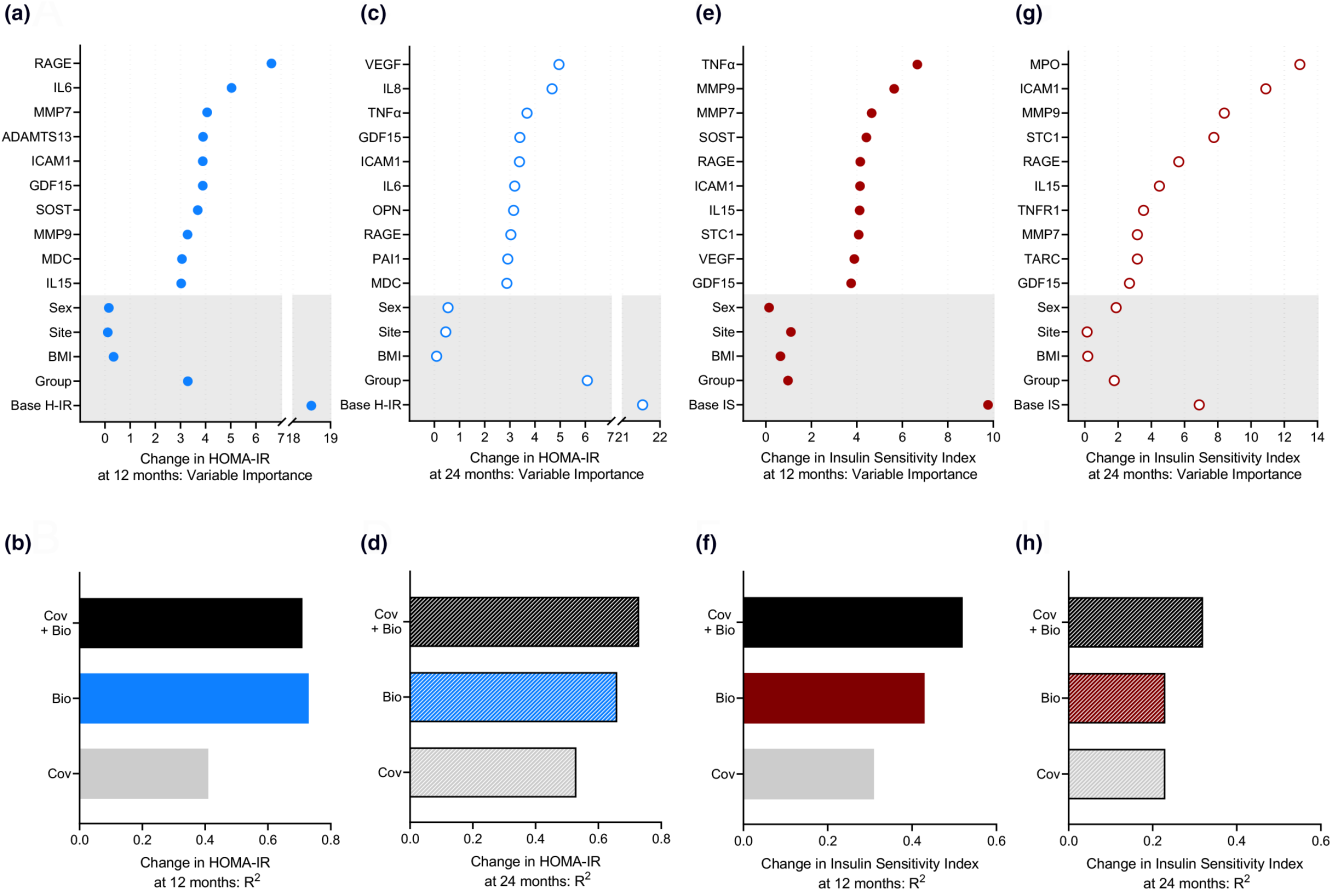
Changes in senescence‐associated biomarkers predict changes in HOMA‐IR and insulin sensitivity index at 12 and 24 months. (a, c) The relative importance of change in the top 10 biomarkers (12 months–baseline and 24 months–baseline) and of site, sex, BMI stratum (normal/overweight), intervention group (AL/CR), and baseline HOMA‐IR (base H‐IR) as determined by GBM to predict changes at 12 months and 24 months in HOMA‐IR. (b, d) The R^2^ of the GBM models—covariates (Cov) (sex, BMI stratum, site, intervention group, and base H‐IR) alone, top 10 biomarkers alone (Bio), or covariates plus top 10 biomarkers (Cov + Bio)—for predicting change in HOMA‐IR at 12 months and 24 months. (e, g) Variable importance summaries and (f, h) the *R* ^2^ of the GBM models for predicting change in the insulin sensitivity index at 12 months and at 24 months using the same covariates as above except for the use of baseline insulin sensitivity index (base IS) in place of base H‐IR. AL, ad libitum; BMI, body mass index; CR, calorie restriction; GBM, gradient boosting machine learning.

Gradient boosting machine learning (GBM) regression modeling identified baseline HOMA‐IR as the most robust predictor of the change in HOMA‐IR at 12 months (Figure 2a). The model also identified the changes in senescence biomarkers as important predictors, with RAGE, IL6, MMP7, ADAMTS13, ICAM1, GDF15, SOST, MMP9, MDC, and IL15 being the top 10. The *R* ^2^ of the model including baseline HOMA‐IR, sex, site, BMI stratum, and intervention group for predicting change in HOMA‐IR at 12 months was 0.41, and markedly increased to 0.71 with the addition of the top 10 biomarkers (Figure 2b).

The GBM model predicting change in HOMA‐IR at 24 months identified baseline HOMA‐IR as the most robust predictor, followed by intervention group (Figure 2c). Changes in the concentration of senescence biomarkers were also identified as important predictors, including 5 of the top 10 that emerged at 12 months (IL6, GDF15, ICAM1, RAGE, and MDC). The *R* ^2^ of the model with only the covariates for predicting change in HOMA‐IR at 24 months was 0.53 and increased to 0.73 with the addition of the top 10 biomarkers (Figure 2d).

As a complementary analysis, we applied GBM regression modeling to identify predictors of change in the insulin sensitivity index, another exploratory metabolic outcome that improved in CR compared to AL participants at both the 12‐ and 24‐month timepoints (Kraus et al., 2019). GBM modeling identified the baseline insulin sensitivity index as the most robust predictor of its change at 12 months (Figure 2e). Changes in the concentrations of senescence biomarkers were also identified as important predictors of change in the insulin sensitivity index at 12 months, with TNFα, MMP9, MMP7, SOST, RAGE, ICAM1, IL15, STC1, VEGF, and GDF15 being the top 10, and of greater significance than sex, site, BMI stratum, or intervention group. The *R* ^2^ of the model including only the covariates for predicting change in the insulin sensitivity index at 12 months was 0.31 and increased to 0.52 with the addition of the top 10 biomarkers (Figure 2f).

The GBM model predicting change in insulin sensitivity index at 24 months identified change in MPO concentration as the most robust predictor (Figure 2g). Changes in the concentration of several other biomarkers were also identified as important predictors, with 7 of the top 10 being the same as at 12 months (ICAM1, MMP9, STC1, RAGE, IL15, MMP7, and GDF15). The *R* ^2^ of the model including only the covariates for predicting change at 24 months in the insulin sensitivity index improved from 0.23 to 0.32 by the addition of the top 10 biomarkers (Figure 2h).

Finally, as an additional analysis we applied GBM regression modeling to identify predictors of the change in resting metabolic rate (RMR) residual, defined as the difference between an individual's RMR measured by indirect calorimetry and RMR predicted from a regression of RMR as a function of fat mass and fat‐free mass. As RMR residual decreased significantly more in CR than AL participants at 12 months but not at 24 months (Ravussin et al., 2015), we focused our analysis on the first timepoint. Baseline RMR residual was the most robust predictor of the change in RMR residual at 12 months (Figure S1a). The changes in senescence biomarkers also emerged as important predictors, with five of the top 10 (ICAM1, IL15, SOST, RAGE, and MMP7) being the same as those identified for change in HOMA‐IR and insulin sensitivity index at 12 months. TNFR2, IL7, activin A, VEGF, and ADAMTS13 completed the top 10, which were all of greater importance than site and intervention group. The R^2^ of the model including only baseline RMR residual, site, and intervention group increased from 0.30 to 0.51 with addition of the top 10 senescence biomarkers (Figure S1b).

### 2.4 Calorie restriction reduces a senescence‐associated gene set in human adipose tissue

**FIGURE 3:**
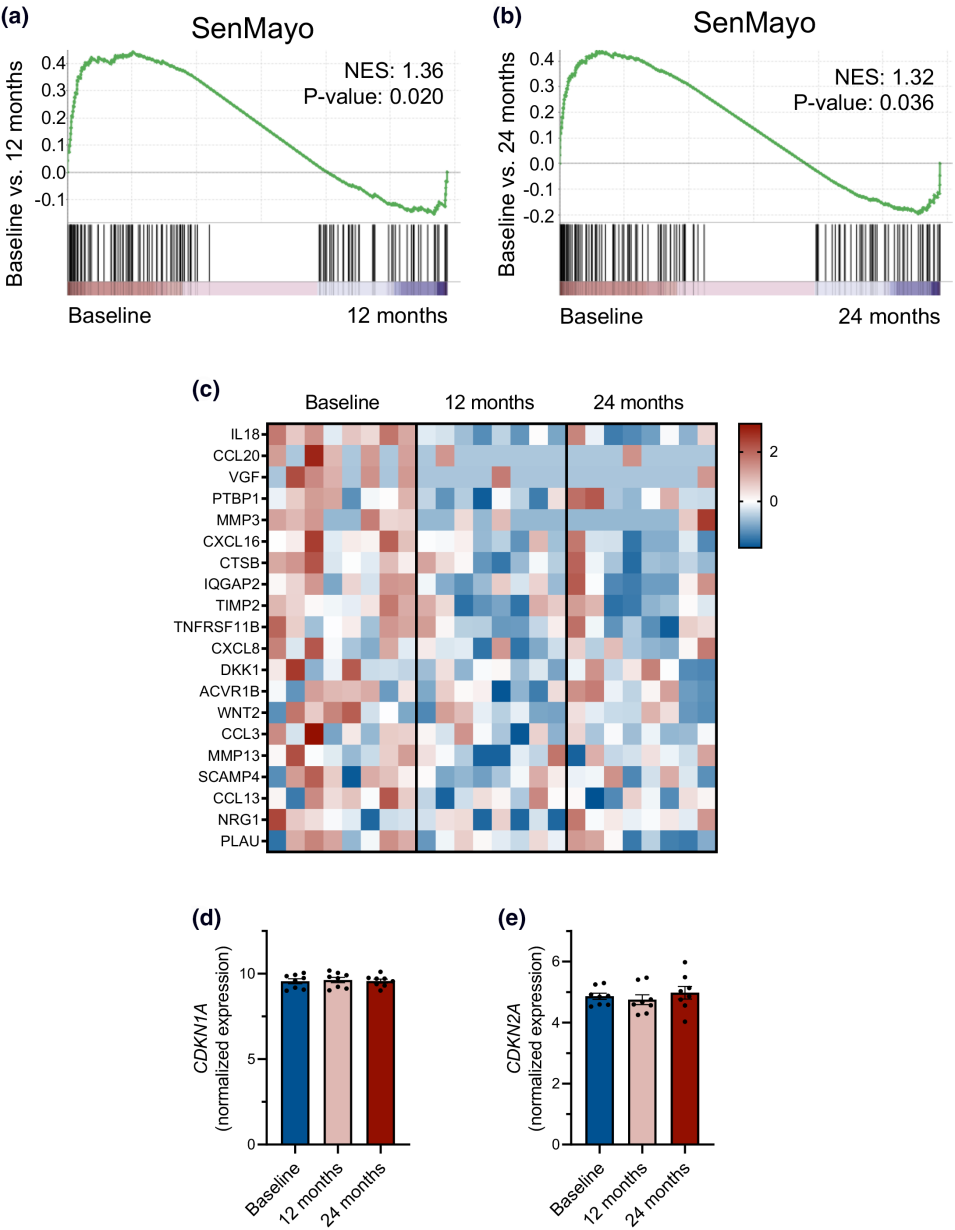
Calorie restriction reduces a senescence‐associated gene set in human adipose tissue. (a, b) GSEA plots of the SenMayo gene set comparing baseline to 12 months and baseline to 24 months using recently published RNA sequencing data derived from the adipose tissue specimens of eight CALERIE™ participants randomized to CR (Spadaro et al., 2022). (c) Heat map with expression of a subset of the genes included in SenMayo at baseline, 12 months, and 24 months. (d, e) Normalized gene expression of *CDKN1A* (*P21*) and *CDKN2A* (*P16*) at baseline, 12 months, and 24 months. CR, calorie restriction; GSEA, gene set enrichment analysis.

To corroborate our data demonstrating a reduction in circulating senescence biomarkers in response to CR, we examined tissue‐level changes in a recently defined gene set of 125 secreted factors, transmembrane proteins, and intracellular proteins centered on cellular senescence and the SASP, named SenMayo (Saul et al., 2022). We used recently published RNA sequencing data derived from baseline, 12‐month, and 24‐month adipose tissue specimens of eight study participants randomized to CR (Spadaro et al., 2022). Through gene set enrichment analysis (GSEA) we observed a significant reduction in SenMayo in response to CR, which is reflected by an enrichment at baseline compared to 12 months (normalized enrichment score [NES]: 1.36, *p*‐value: 0.020) and at baseline compared to 24 months (NES: 1.32, *p*‐value: 0.036) (Figure 3a–c).

We note that we did not detect a significant change by RNA sequencing in expression of *CDKN1A* (*P21*) or *CDKN2A* (*P16*) (Figure 3d,e), two prototypical markers of cellular senescence (Hernandez‐Segura et al., 2018). This may be related to their variable and low levels of expression, particularly in younger and healthier adults. For these reasons, neither *CDKN1A* nor *CDKN2A* are included in the SenMayo gene set.

## 3 DISCUSSION

In this study we found that 2 years of moderate CR with adequate nutrient intake compared to AL significantly decreased the circulating levels of several senescence‐associated biomarkers in healthy, young to middle‐aged individuals without obesity. A greater number of biomarkers were modulated at 12 months than at 24 months, but PAI1, PARC, TARC, and TNFR1 were lower in CR participants at both timepoints. Using a machine learning approach, we observed that the changes in several biomarkers were important predictors of the change in CALERIE™ metabolic outcomes, including HOMA‐IR, insulin sensitivity index, and RMR residual. Our results advance the mechanistic understanding of CR and suggest a potential link between cellular senescence and metabolic health in humans.

Previous findings from the CALERIE™ study evidenced a reduction in systemic markers of inflammation, such as C‐reactive protein, in participants randomized to the CR intervention (Kraus et al., 2019; Ravussin et al., 2015). Low grade “sterile” systemic inflammation is considered a risk factor for several age‐related chronic diseases and, although its pathogenesis is multifactorial, senescent cells through their SASP are a plausible source of proinflammatory molecules (Ferrucci & Fabbri, 2018). Unfortunately, none of the SASP components studied to date is unique to senescent cells and, certainly, the secretome of other cell types may be affected by CR. We note, however, that the circulating biomarkers assessed in this study were based on an expanded panel of biologically diverse senescence‐related proteins we previously developed by reviewing the literature, screening their abundance in in vitro models of senescence, and demonstrating detectability in human blood in contexts associated with increased senescent cell burden (Aversa et al., 2023; Fielding et al., 2022; Schafer et al., 2020). In addition, our in silico analysis of an RNA sequencing data set derived from the adipose tissue specimens of a small subset of CALERIE™ participants randomized to CR (Spadaro et al., 2022) demonstrated a significant reduction in the SenMayo gene set at both 12 and 24 months compared to baseline. At this time, however, we cannot disentangle whether the reduced levels in circulating senescence‐related biomarkers observed in response to CR reflect reduced senescent cell accumulation, increased clearance, or inhibition of their SASP. It is also not clear what organs are the main targets of the intervention. Results from our in silico analysis would suggest that CR may target senescent cells in adipose tissue, but the small sample size limits the generalizability of these results. Moreover, the previous analysis of this RNA sequencing dataset evidenced a reduction in macrophage‐specific transcripts after deconvolution at both 12 and 24 months suggesting again that CR can also exert its anti‐inflammatory effects by targeting other cells types such as resident immune populations in adipose tissue (Spadaro et al., 2022). Senescent cells, however, can also recruit and activate immune cells through their secretome (Kale et al., 2020) therefore our data are consistent with previous observations.

Among the biomarkers significantly modulated by CR at 12 months, only SOST (sclerostin) was observed to significantly increase. Although we previously reported that SOST is especially produced by senescent endothelial cells in vitro (Schafer et al., 2020), this negative regulator of bone formation is a protein released predominantly by osteocytes (Delgado‐Calle et al., 2017) and previously reported to increase with weight loss in older adults with obesity when diet was not combined with exercise (Armamento‐Villareal et al., 2012). Prior results from the CALERIE™ study evidenced changes in bone turnover markers (i.e., increase in C‐telopeptide and tartrate‐resistant acid phosphatase at 12 months, and decrease in bone‐specific alkaline phosphatase at 12 and 24 months), along with a mild but statistically significant reduction in bone mineral density in CR participants (Villareal et al., 2016); therefore, it is possible that the SOST increase observed here may reflect alterations in bone metabolism.

The circulating levels of RAGE (receptor for advanced glycation end‐products) also significantly increased in CR compared to AL participants at 24 months. RAGE is a member of the immunoglobulin superfamily of receptors that exists in a membrane‐bound form as well as in a soluble form, which acts as a decoy receptor (Bongarzone et al., 2017). A nuclear isoform of RAGE has also been identified and implicated in DNA repair. Interestingly, RAGE knockout mice develop lung fibrosis and display markers of cellular senescence (Kumar et al., 2017), but the direct effects of senescence on circulating RAGE abundance still need to be carefully investigated.

The CALERIE™ study was designed primarily to assess the potential of long‐term, moderate CR to induce metabolic adaptation as defined by a reduction in core body temperature and RMR residual (Rochon et al., 2011). Metabolic slowing has in fact been proposed as one of the potential mechanisms underlying the anti‐aging effects of CR as it may reduce oxidative stress and damage (Redman et al., 2018). As reported previously, core body temperature was not changed meaningfully, while RMR residual decreased significantly in the CR compared to the AL group at 12 but not 24 months (Ravussin et al., 2015). Using machine learning, we identified that changes in several senescence biomarkers were important predictors of this metabolic adaptation. Indeed, baseline RMR residual, site, and intervention group explained 30% of variance in change in RMR residual at 12 months when analyzed alone and 51% of the variance when combined with the top 10 biomarkers. Moreover, we observed that changes in several senescence‐related proteins at 12 months were also predictive of changes in HOMA‐IR and insulin sensitivity index, with the models including the 10 top biomarkers outperforming the predictive ability of the models including only the clinical and demographic variables. Of note, 5 biomarkers (ICAM1, IL15, SOST, RAGE, and MMP7) emerged as important predictors of change for all three metabolic outcomes at 12 months. In addition, for both HOMA‐IR and insulin sensitivity index, several biomarkers persisted among the top 10 in the models predicting change at 24 months. Importantly, the models predicting change in HOMA‐IR displayed a consistently higher predictive ability at both 12 and 24 months. Together, these results suggest that changes in plasma levels of several senescence‐related proteins are associated with measures of metabolic health, and are in agreement with preclinical studies that have implicated senescence in the pathogenesis of insulin resistance and showed that behavioral (exercise), genetic or pharmacological clearance of senescent cells in obese mice improves insulin sensitivity (Palmer et al., 2019; Schafer et al., 2016; Wang et al., 2022; Wiley & Campisi, 2021). We acknowledge that the current analysis does not allow us to establish whether changes in senescence biomarkers and metabolic parameters in humans simply paralleled each other or were causally linked.

Results from this study are complementary to previous analyses of the CALERIE™ study and further highlight the potential of nutritional interventions to counter biological aging. Participants in the CALERIE™ study were healthy, young to middle‐aged individuals of normal weight to moderately overweight, yet several senescence‐related proteins previously associated with chronological age and with clinical measures of biological age and functional limitations (Aversa et al., 2023; Fielding et al., 2022; Schafer et al., 2020; Tanaka et al., 2018, 2020) were quantifiable in their plasma and were, at least in part, longitudinally reduced by the CR intervention. For example, PARC, TARC, TNFR1, TNFR2, and VEGF were reduced by the CR intervention and exhibited positive associations with chronological age in at least two of three prior studies in which they were examined (Schafer et al., 2020; Tanaka et al., 2018, 2020).

Consistent with our findings, a previous intent‐to‐treat analysis of the CALERIE™ study suggested that CR can slow biological aging as measured by two different algorithms (Klemera–Doubal Method Biological Age and homeostatic dysregulation) (Belsky et al., 2017). Moreover, another recent study reported that the CR intervention in CALERIE™ participants slowed the pace of aging (that is, change in biological age per chronological year) as measured by the DunedinPACE algorithm of blood DNA methylation data (Waziry et al., 2023). Although it is currently unknown whether lower markers of biological aging are sustained over time and if there are long‐term implications, a follow‐up of the CALERIE™ study supported by the National Institute on Aging (R01AG071717) is ongoing and will investigate the legacy effects of the 2‐year CR intervention on healthspan and hallmarks of aging (NCT05651620).

Major strengths of this study include the use of biospecimens and clinical data collected in a rigorous manner from participants in CALERIE™, the first randomized controlled trial of CR in humans without obesity. These attributes of the parent study facilitated the unbiased analysis of how CR, arguably the most studied intervention targeting aging, influences the circulating levels of a biologically diverse set of senescence‐associated proteins and their relationship with metabolic health outcomes in humans. However, several limitations need to be considered when interpreting the results. First, none of the circulating biomarkers measured in the present study unequivocally and universally detect senescent cells. While this challenge is generally overcome in preclinical studies by systematically assessing multiple core properties of senescent cells in several tissues, this approach is not feasible in the context of a clinical study. Our in silico analysis provided a proof of concept of the potential CR‐mediated senotherapeutic effect on adipose tissue, but additional assessments are needed to further explore this hypothesis. Second, while the GBM models suggested that the changes in several senescence‐related proteins were associated with changes in HOMA‐IR, insulin sensitivity index, and RMR residual, our findings do not imply causation. Third, participants in the CALERIE™ study were 20–50 years of age and were recruited based on stringent pre‐defined eligibility criteria, which may limit the generalizability of the results to the general population.

In conclusion, our results show that 2 years of moderate CR with adequate nutrient intake reduces biomarkers of cellular senescence in healthy young to middle‐aged humans without obesity. Additional research is needed to fully elucidate the senotherapeutic implications of CR, validate the biomarkers signatures that emerged as predictive of metabolic health outcomes, and determine the generalizability and durability of the observed effects. Our data further highlight the impact of lifestyle factors on fundamental mechanisms of aging.

## 4 METHODS

### 4.1 Study participants

Plasma samples and clinical data were obtained from participants to the CALERIE™ phase 2 study, described in detail elsewhere (Ravussin et al., 2015). Briefly, the CALERIE™ phase 2 study was a multicenter, randomized controlled trial which, between May 2007 and November 2012, enrolled healthy, normal weight, and slightly overweight men (aged 21–50 years) and premenopausal women (aged 21–47 years), and randomized them to 25% CR or an AL diet with an allocation ratio of 2:1. We included in our analyses 199 participants (CR = 128, AL = 71) who had stored plasma specimens available from at least two of the three timepoints.

The study protocol (NCT00427193) was approved by the institutional review boards at all participating clinical centers (Washington University School of Medicine, St Louis, MO; Pennington Biomedical Research Center, Baton Rouge, LA; Tufts University, Boston, MA), and the coordinating center (Duke University, Durham, NC). All study participants provided written informed consent and study oversight was provided by a Data and Safety Monitoring Board.

The present analyses were conducted on coded data with no personal identifiers and deemed exempt from the requirement for Institutional Review Board approval (45 CFR 46.101, item 4).

### 4.2 Measurement of circulating senescence biomarkers

The concentrations of protein biomarkers were quantified in plasma samples collected at baseline, 12‐ and 24‐month follow‐up timepoints using commercially available multiplex magnetic bead‐based immunoassays (R&D Systems) on the Luminex xMAP multianalyte profiling platform and analyzed on a MAGPIX System (Merck Millipore). All assays were performed according to the manufacturer's protocols. Twenty‐seven biomarkers were quantified with this method: ADAMTS13, eotaxin, Fas, GDF15, ICAM1, IL6, IL7, IL8, IL15, MDC, MMP1, MMP2, MMP7, MMP9, MPO, OPN, PAI1, PARC, RAGE, RANTES, SOST, STC1, TARC, TNFα, TNFR1, TNFR2, and VEGF. Activin A concentration was determined by a Quantikine ELISA Kit (R&D Systems) according to the manufacturer's instructions. In instances where a biomarker was below the limit of detection in a participant's sample, a value of half of the lowest measured value for that analyte was assigned. Assay performance characteristics are reported in Table S3.

### 4.3 Metabolic health outcomes

We used HOMA‐IR, insulin sensitivity index, and RMR residual as metabolic health outcomes.

The HOMA‐IR was calculated as fasting glucose (mmol/L) × fasting insulin (mIU/L)/22.5 (Matthews et al., 1985). The insulin sensitivity index was calculated as 1 divided by fasting insulin concentration (mIU/L) (Lee et al., 2011). RMR residual, defined as the difference between an individual's RMR measured by indirect calorimetry and RMR predicted from a regression of RMR as a function of fat mass and fat‐free mass, was calculated from the corresponding measures performed at baseline, 12 months, and 24 months as previously described in detail (Ravussin et al., 2015).

### 4.4 RNA sequencing analysis

The fastq files were obtained from Synapse ID syn23667189 after permission from Spadaro et al. (2022) and were mapped to the human reference genome hg38. Subsequent analysis was performed as previously described (Saul et al., 2022; Spadaro et al., 2022). Briefly, significantly differentially regulated genes were selected by a Benjamini–Hochberg adjusted *p* value < 0.05 and log2‐fold changes above 0.5 or below −0.5. The GSEA (Subramanian et al., 2005) was performed with default settings (1000 permutations for gene sets, Signal2Noise metric for ranking genes). The heatmap was designed with GraphPad Prism (Prism software, Version 9.0, San Diego, CA).

### 4.5 Statistical methods

As for the original trial, statistical analysis was performed following intention‐to‐treat (ITT) principles. Baseline characteristics were assessed using the Wilcoxon rank‐sum test and the chi‐squared test. Prior to analysis, the biomarkers were log transformed and then standardized to have a mean of 0 and SD of 1. Group (AL and CR) differences in senescence biomarkers over time were individually assessed using mixed effects repeated measures analysis after adjusting for study site, sex, BMI strata, the baseline biomarker value. Predicted mean changes ± standard errors were calculated, along with between group differences at each timepoint.

In order to assess the combined effect of the biomarkers beyond the intervention on the endpoints of interest, GBM models were fit predicting change in HOMA‐IR, change in the insulin sensitivity index, and change in RMR residual at 12 months and at 24 months using the change in biomarker values. For change in HOMA‐IR and change in insulin sensitivity index, the baseline covariates included study site, sex, BMI strata, intervention group and baseline HOMA‐IR or baseline insulin sensitivity index, respectively. For change in RMR residual, baseline covariates included study site, intervention group, and baseline RMR residual. Models were fit using baseline covariates alone, biomarkers alone, covariates plus biomarkers, top 10 biomarkers alone, and top 10 biomarkers plus covariates. Analyses were performed using R 4.2.2.

## AUTHOR CONTRIBUTIONS

Conception or design of the work: Zaira Aversa, Elizabeth J. Atkinson, and Nathan K. LeBrasseur. Acquisition, analysis, or interpretation of data for the work: Zaira Aversa, Thomas A. White, Amanda A. Heeren, Cassondra A. Hulshizer, Dominik Saul, Xu Zhang, Anthony J. A. Molina, Leanne M. Redman, Corby K. Martin, Susan B. Racette, Kim M. Huffman, Manjushri Bhapkar, Sundeep Khosla, Sai Krupa Das, Roger A. Fielding, Elizabeth J. Atkinson, and Nathan K. LeBrasseur. Drafting of the work: Zaira Aversa, Elizabeth J. Atkinson, and Nathan K. LeBrasseur. Critical revision: Zaira Aversa, Thomas A. White, Amanda A. Heeren, Cassondra A. Hulshizer, Dominik Saul, Xu Zhang, Anthony J. A. Molina, Leanne M. Redman, Corby K. Martin, Susan B. Racette, Kim M. Huffman, Manjushri Bhapkar, Sundeep Khosla, Sai Krupa Das, Roger A. Fielding, Elizabeth J. Atkinson, and Nathan K. LeBrasseur.

## CONFLICT OF INTEREST STATEMENT

NKL and Mayo Clinic have intellectual property related to this work licensed to a commercial entity. This research has been reviewed by the Mayo Clinic Conflict of Interest Review Board and is being conducted in compliance with Mayo Clinic Conflict of Interest policies.

## Supporting information


Figure S1:
Table S1:
Table S2:
Table S3:

## Data Availability

All clinical data were obtained from the CALERIE™ phase 2 study. Data related to senescence biomarkers are available from the corresponding author upon reasonable request. For RNA sequencing analysis the fastq files were obtained from Synapse ID syn23667189 after permission from Spadaro et al. (2022).
