# Supplementary material for: Calorie restriction reduces biomarkers of cellular senescence in humans
Source: Aging Cell. 2023 Nov 14;23(2):e14038. doi: 10.1111/acel.14038 (PMC10861196; doi:10.1111/acel.14038)
Supplement: Supplementary file 1 — Figure S1: Table S1: Table S2: Table S3: [file ACEL-23-e14038-s001.pdf]

SUPPLEMENTAL INFORMATION

Calorie Restriction Reduces Biomarkers of Cellular Senescence in Humans

Zaira Aversa, Thomas A. White, Amanda A. Heeren,

Cassondra A. Hulshizer, Dominik Saul, Xu Zhang, Anthony J. A. Molina, Leanne M. Redman,

Corby K. Martin, Susan B. Racette, Kim M. Huffman, Manjushri Bhapkar, Sundeep Khosla,

Sai Krupa Das, Roger A. Fielding, Elizabeth J. Atkinson,

Nathan K. LeBrasseur

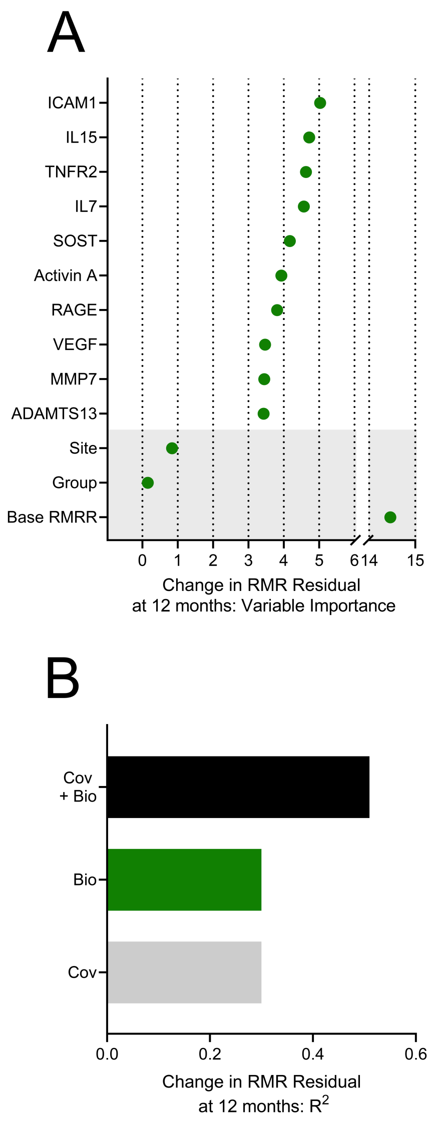

Supplementary Figure S1: Changes in senescence-associated biomarkers predict changes in RMR residual at 12 months. (A) The relative importance of change in the top ten biomarkers (12 months – baseline) and of site, intervention group (AL/CR), and baseline resting metabolic rate residual (base RMRR) as determined by gradient boosting modeling (GBM) to predict changes at 12 months in resting metabolic rate (RMR) residual. (B) The R^2 of the GBM models — covariates (Cov) (site, intervention group, and base RMRR) alone, top 10 biomarkers alone (Bio), or covariates plus top 10 biomarkers (Cov + Bio) — for predicting change in RMR residual.

Supplementary Table S1: List of the Proteins Measured in the Plasma of CALERIE™ Participants

Protein name	Protein full name	Alias
Activin A	Activin A	INHBA
ADAMTS13	A disintegrin and metalloproteinase with thrombospondin motifs 13	VWFCP
Eotaxin	Eotaxin	CCL11
Fas	Tumor necrosis factor receptor superfamily member 6	APT1, TNFRSF6
GDF15	Growth/differentiation factor 15	MIC1, NAG1, NRG1
ICAM1	Intercellular adhesion molecule 1	CD54
IL6	Interleukin 6	IFNB2
IL7	Interleukin 7	
IL8	Interleukin 8	CXCL8
IL15	Interleukin 15	
MDC	Macrophage-derived chemokine	CCL22, SCYA22
MMP1	Matrix metalloproteinase 1	Interstitial collagenase
MMP2	Matrix metalloproteinase 2	CLG4A
MMP7	Matrix metalloproteinase 7	Matrilysin
MMP9	Matrix metalloproteinase 9	CLG4B
MPO	Myeloperoxidase	
OPN	Osteopontin	SPP1
PAI1	Plasminogen activator inhibitor 1	SERPINE1, PLANH1
PARC	Pulmonary and activation-regulated chemokine	CCL18
RAGE	Advanced glycosylation end product-specific receptor	
RANTES	Regulated on Activation, Normal T Cell Expressed and Secreted	CCL5, SCYA5
SOST	Sclerostin	DAND6
STC1	Stanniocalcin 1	
TARC	Thymus and activation-regulated chemokine	CCL17, SCYA17
TNFα	Tumor necrosis factor alpha	TNFSF2
TNFR1	Tumor necrosis factor receptor 1	TNFRSF1A, CD120a
TNFR2	Tumor necrosis factor receptor 2	TNFRSF1B
VEGF	Vascular endothelial growth factor	VPF

Supplementary Table S2: Senescence-associated Biomarkers at Baseline, and Changes from Baseline at 12 and 24 Months

	Ad libitum group	Calorie restriction group	Between-group p-value
**Activin A**			
Baseline	0.06 (0.09)	0.12 (0.09)	
Change at month 12	0.05 (0.09)	-0.14 (0.07)	0.083
Change at month 24	0.02 (0.09)	-0.04 (0.07)	0.563
**ADAMTS13**			
Baseline	0.16 (0.13)	-0.26 (0.09)	
Change at month 12	0.03 (0.06)	0.09 (0.04)	0.37
Change at month 24	0.02 (0.06)	0.04 (0.04)	0.71
**Eotaxin**			
Baseline	-0.09 (0.14)	0.14 (0.07)	
Change at month 12	0.09 (0.09)	0.05 (0.07)	0.743
Change at month 24	-0.09 (0.09)	0.11 (0.07)	0.064
**Fas**			
Baseline	-0.02 (0.11)	0.01 (0.09)	
Change at month 12	0.01 (0.06)	0.01 (0.05)	0.994
Change at month 24	0.07 (0.06)	0.02 (0.05)	0.517
**GDF15**			
Baseline	-0.02 (0.11)	-0.16 (0.09)	
Change at month 12	0.15 (0.08)	0.07 (0.06)	0.405
Change at month 24	0.16 (0.08)	0.07 (0.06)	0.348
**ICAM1**			
Baseline	-0.04 (0.11)	0.06 (0.09)	
Change at month 12	0.03 (0.03)	-0.01(0.02)	0.213
Change at month 24	**0.04 (0.03)**	**-0.05 (0.02)**	**0.008**
**IL6**			
Baseline	0.19 (0.10)	-0.02 (0.09)	
Change at month 12	-0.17 (0.12)	-0.09 (0.09)	0.518
Change at month 24	0.01 (0.11)	0.05 (0.09)	0.725
**IL7**			
Baseline	0.33 (0.11)	0.23 (0.09)	
Change at month 12	**0.21 (0.12)**	**-0.13 (0.09)**	**0.013**
Change at month 24	-0.10 (0.11)	-0.19 (0.09)	0.544
**IL8**			
Baseline	0.12 (0.10)	0.16 (0.07)	
Change at month 12	0.10 (0.11)	-0.05 (0.08)	0.266
Change at month 24	-0.08 (0.11)	-0.02 (0.09)	0.641
**IL15**			
Baseline	0.15 (0.11)	-0.18 (0.10)	
Change at month 12	0.03 (0.06)	0.00 (0.05)	0.625
Change at month 24	0.06 (0.06)	0.10 (0.05)	0.598
**MDC**			
Baseline	0.17 (0.11)	0.02 (0.09)	
Change at month 12	0.00 (0.08)	-0.04 (0.06)	0.694
Change at month 24	0.02 (0.08)	-0.10 (0.06)	0.208

	Ad libitum group	Calorie restriction group	Between-group p-value
**MMP1**			
Baseline	0.00 (0.12)	0.21 (0.09)	
Change at month 12	**0.20 (0.10)**	**-0.05 (0.08)**	**0.035**
Change at month 24	0.04 (0.10)	-0.13 (0.08)	0.157
**MMP2**			
Baseline	-0.22 (0.17)	0.09 (0.07)	
Change at month 12	-0.07 (0.11)	-0.01 (0.08)	0.639
Change at month 24	-0.03 (0.11)	0.10 (0.08)	0.312
**MMP7**			
Baseline	-0.42 (0.13)	-0.29 (0.10)	
Change at month 12	0.20 (0.10)	0.22 (0.07)	0.817
Change at month 24	0.14 (0.10)	0.20 (0.07)	0.589
**MMP9**			
Baseline	-0.02 (0.11)	0.27 (0.09)	
Change at month 12	-0.13 (0.12)	-0.06 (0.09)	0.612
Change at month 24	-0.02 (0.12)	-0.08 (0.09)	0.656
**MPO**			
Baseline	0.02 (0.10)	0.16 (0.09)	
Change at month 12	**0.19 (0.11)**	**-0.16 (0.08)**	**0.01**
Change at month 24	0.06 (0.11)	0.02 (0.09)	0.78
**OPN**			
Baseline	-0.04 (0.10)	-0.15 (0.10)	
Change at month 12	0.07 (0.08)	0.12 (0.06)	0.589
Change at month 24	0.12 (0.08)	0.09 (0.06)	0.738
**PAI1**			
Baseline	0.50 (0.11)	0.22 (0.08)	
Change at month 12	**0.38 (0.11)**	**-0.28 (0.08)**	**<0.001**
Change at month 24	**0.12 (0.11)**	**-0.26 (0.08)**	**0.003**
**PARC**			
Baseline	0.16 (0.12)	0.05 (0.09)	
Change at month 12	**0.07 (0.05)**	**-0.11 (0.04)**	**0.006**
Change at month 24	**0.11 (0.05)**	**-0.12 (0.04)**	**<0.001**
**RAGE**			
Baseline	-0.04 (0.12)	-0.07 (0.09)	
Change at month 12	-0.02 (0.06)	0.07 (0.04)	0.149
Change at month 24	**-0.05 (0.06)**	**0.11 (0.04)**	**0.024**
**RANTES**			
Baseline	0.27 (0.12)	0.22 (0.08)	
Change at month 12	**0.27 (0.11)**	**-0.09 (0.08)**	**0.005**
Change at month 24	-0.14 (0.11)	-0.21 (0.08)	0.634
**SOST**			
Baseline	-0.02 (0.13)	-0.02 (0.08)	
Change at month 12	**-0.12 (0.07)**	**0.11 (0.06)**	**0.012**
Change at month 24	0.00 (0.07)	0.11 (0.06)	0.207
**STC1**			
Baseline	-0.10 (0.12)	0.08 (0.09)	
Change at month 12	-0.02 (0.08)	0.00 (0.06)	0.815
Change at month 24	0.03 (0.07)	0.03 (0.06)	0.990

	Ad libitum group	Calorie restriction group	Between-group p-value
**TARC**			
Baseline	0.33 (0.10)	0.04 (0.10)	
Change at month 12	**0.25 (0.10)**	**-0.12 (0.08)**	**0.002**
Change at month 24	**0.12 (0.10)**	**-0.14 (0.08)**	**0.035**
**TNFα**			
Baseline	-0.02 (0.10)	0.13 (0.09)	
Change at month 12	0.04 (0.08)	-0.04 (0.06)	0.374
Change at month 24	0.02 (0.08)	-0.07 (0.06)	0.328
**TNFR1**			
Baseline	-0.05 (0.11)	0.06 (0.09)	
Change at month 12	**0.09 (0.06)**	**-0.08 (0.04)**	**0.019**
Change at month 24	**0.19 (0.06)**	**-0.05 (0.05)**	**<0.001**
**TNFR2**			
Baseline	-0.12 (0.12)	0.13 (0.09)	
Change at month 12	0.07 (0.07)	-0.08 (0.05)	0.074
Change at month 24	**0.13 (0.07)**	**-0.11 (0.05)**	**0.003**
**VEGF**			
Baseline	0.02 (0.17)	0.21 (0.07)	
Change at month 12	**0.21 (0.12)**	**-0.18 (0.09)**	**0.005**
Change at month 24	-0.05 (0.11)	-0.09 (0.09)	0.775

Baseline values are the observed mean (SE); change scores are the least-squares adjusted means (SE) from the intention-to-treat repeated measures analysis. Between-group p-value tests for a significant between-group difference in the change score at the timepoint.

Supplementary Table S3. Assay performance characteristics, including measurement range, sensitivity, and the coefficient of variation (CV) for biomarkers of cellular senescence.

**Biomarker**	*Range (pg/ml)*	*Sensitivity (pg/ml)*	*CV(%)*
Activin A	15.6-1000	7.85	8.51
ADAMTS13	16000-3900000	1270	13.99
Eotaxin	32.3-23580	1.81	10.42
Fas	144-35000	3.2	12.99
GDF15	18.5-4500	1.2	8.22
ICAM1	7000-1700000	87.9	9.88
IL6	0.7-2800	0.135	9.08
IL7	0.9-3620	0.140	10.31
IL8	0.879-3600	0.07	9.48
IL15	0.6-2350	0.167	14.28
MDC	53.5-13000	8.5	8.21
MMP1	49.4-12000	2.7	11.72
MMP2	276-67000*	108	18.22
MMP7	226-55000	23.2	13.85
MMP9	123-30000*	13.6	5.57
MPO	123-30000*	26.2	7.75
OPN	1440-350000	413	11.88
PAI1	18.1-4400*	0.7	7.01
PARC	18.5-4500*	0.3	5.74
RAGE	123-30000	7.2	6.27
RANTES	20.6-5000*	1.8	8.77
SOST	10.3-2500	7	8.51
STC1	123-30000	11.6	9.73
TARC	103-25000	8.9	10.81
TNFα	0.757-3100	0.54	8.17
TNFR1	49.4-12000	41	7.11
TNFR2	11.1-2700	0.5	12.40
VEGF	7-4280	1.17	8.94

* Denotes biomarkers analyzed in plasma samples at 1:100 dilution.
All other biomarkers analyzed in plasma samples at 1:2 dilution.
